# Recurrent Mental Cutaneous Metastases Secondary to Bilateral Renal Cell Carcinoma

**DOI:** 10.7759/cureus.103673

**Published:** 2026-02-15

**Authors:** Jessica L Hoffman, Chase E Green, Monica Botros, Javier Corral

**Affiliations:** 1 Internal Medicine, Texas Tech University Health Sciences Center El Paso, El Paso, USA; 2 Hematology and Oncology, Texas Tech University Health Sciences Center El Paso, El Paso, USA

**Keywords:** bilateral rcc, bladder metastasis, cutaneous metastasis, metastatic rcc, renal cell carcinoma, supportive and palliative care

## Abstract

Renal cell carcinoma (RCC) is a common cancer of the urinary tract that continues to increase in incidence. With advances in imaging, RCC is being detected more frequently and at earlier stages. However, metastatic disease remains a common concern, resulting in worse prognoses. Common sites of metastases include the lungs, bone, lymph nodes, liver, brain, and adrenal glands. Bladder metastases are rare, with fewer than a hundred cases reported in the literature. Cutaneous metastases are also uncommon but have been reported. The case presented is a unique account of bilateral RCC with the development of both bladder and cutaneous metastases in the setting of prolonged survival. One treatment option for cutaneous metastases is surgical resection, as described in this case. However, based on the refractory course observed in this case, surgical resection alone may be insufficient in preventing recurrence, underscoring the need for future research investigating multimodal approaches such as combination therapy with radiation or immunotherapy. This patient went on to receive hospice care, further highlighting the relationship between metastatic disease and worsening prognoses.

## Introduction

Renal cell carcinoma (RCC) accounts for about 2% of global cancer diagnoses and deaths, with a significant burden affecting the United States (US) and the developing world [1]. Annual RCC incidence for the US in 2025 is estimated to exceed 80,000 new cases, resulting in over 14,000 deaths [2]. Treatment options depend on the extent and characteristics of the disease. Surgical resection is common for solitary tumors, while metastatic disease is now frequently targeted with immunotherapies such as aldesleukin (IL-2) [2]. Treatment challenges include resistance to chemotherapy, radiation, and immunotherapies. 

Bilateral RCC occurs when both kidneys exhibit evidence of disease. Subtypes include synchronous bilateral RCC, in which the disease appears simultaneously, and metachronous bilateral RCC, which involves development in the contralateral kidney six months to a year after initial presentation. The incidence of bilateral RCC is low, accounting for less than 5% of all RCC cases, but when present, it is associated with worse outcomes [3].

With advancements in imaging, early detection of RCC has increased. However, distant metastases remain a concern in up to 30% of cases, significantly worsening prognoses. Common sites for RCC metastases include the lung, bone, lymph nodes, liver, brain, and adrenal glands [4]. Cutaneous metastases are rare and thought to occur through hematogenous dissemination after initial lymphatic spread. Cutaneous metastases usually result in poor outcomes, possibly due to this extensive spread [5]. Bladder metastases are also rare, with less than a hundred cases reported in the literature. The mechanism of bladder metastasis secondary to RCC is less clear, but current hypotheses include hematologic spread and the drop metastasis theory. This theory suggests bladder metastases occur when RCC cells enter the collecting system and seed the bladder [6].

In this report, we present a rare case of a previously resected bladder metastasis and recurrent cutaneous metastases secondary to bilateral RCC. This report aims to increase awareness of possible yet rare metastatic manifestations of RCC.

## Case presentation

A 70-year-old male with a past medical history of schizophrenia, hypertension, and bilateral RCC presented with an extensive, bleeding, necrotic anterior chin mass suspicious for cutaneous metastasis.

The patient was initially diagnosed with left RCC in 2019, stage T3aNxMx, more than five years before presentation. Radical left nephrectomy was curative at that time, and pathology demonstrated clear cell RCC. In 2021, the patient was found to have a metachronous right renal mass concerning for primary RCC versus metastasis, in addition to a bladder mass concerning for metastasis. Partial right nephrectomy was used to remove the right renal mass, while transurethral resection of bladder tumor (TURBT) was used to remove a 3 cm tumor in the superior anterior bladder. Immunohistology staining of the bladder mass confirmed metastatic clear cell RCC with positive carbonic anhydrase IX** **and negative GATA binding protein 3 markers. The patient received immunotherapy, but his disease continued to progress.

Per the patient's report, his chin mass first appeared more than a year before presentation. The lesion had been surgically removed twice: first one year before presentation and then again four months after, indicating fast recurrence.

**Figure 1:**
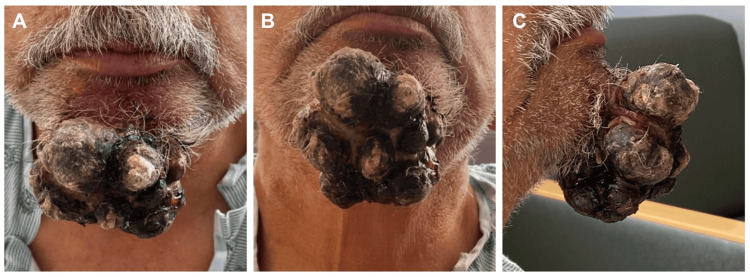
Mental mass prior to surgical resection A: frontal view B: upward chin tilt to show the inferior extent of the lesion C: lateral view highlighting the anterior projection

**Figure 2:**
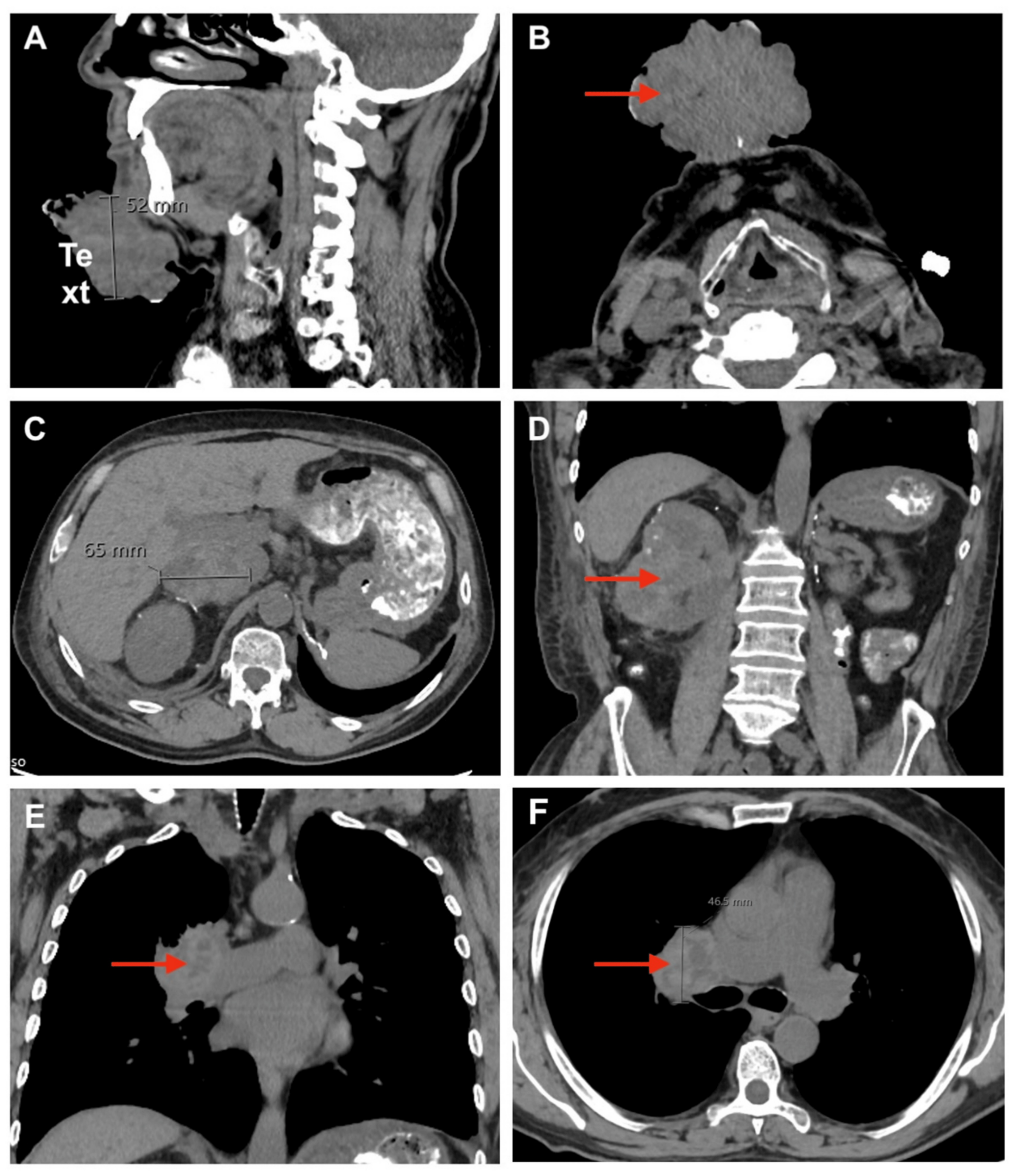
Computed tomography images obtained on admission A: sagittal view of fungating, ulcerated superficial mass arising from the skin overlying the mental protuberance without evidence of deep extension B: axial view of the same mental mass C: axial image demonstrating a grossly abnormal right kidney suspicious for recurrent neoplasm D: coronal view of the same grossly abnormal right kidney E: coronal image of the thorax showing a pulmonary nodule consistent with widespread metastases F: axial image of the thorax showing a pulmonary nodule consistent with widespread metastases

On admission, a physical exam revealed a large, multilobulated, necrotic chin mass (Figure 1). Labs showed normocytic anemia, non-anion gap metabolic acidosis, and stage five chronic kidney disease. Electrolyte abnormalities included hyponatremia, hyperkalemia, hypocalcemia, and hyperchloremia. Magnesium and phosphate were within normal limits. Urinalysis was unremarkable. The neck soft tissue CT showed a fungating, ulcerated superficial mass measuring 73x58x52 mm projecting from the skin overlying the mental protuberance without evidence of deep extension (Figure 2). CT of the abdomen revealed a grossly abnormal right kidney suspicious of recurrent neoplasm and a large right adrenal mass with perinephric soft tissue masses extending into the abdominal wall, concerning for metastatic disease. CT of the thorax showed multiple bilateral pulmonary nodules, further confirming widespread metastatic disease. 

The patient expressed a significant desire for palliative removal of the chin mass. The oral and maxillofacial surgeon excised the mass using radical resection and adjacent tissue transfer via rotation flap. Pathology confirmed morphological features of metastatic RCC with apparent clear cell features. Unfortunately, the course was complicated by wound dehiscence and a positive culture for E. Coli and Proteus mirabilis, which required surgical debridement. The patient then opted for hospice care due to the extensive nature of his cancer.

## Discussion

Cutaneous metastases secondary to RCC are uncommon but reported entities. Incidence is estimated at 3.3% and parallels total RCC incidence by sex [7]. Common cutaneous locations include the scalp, chest, and abdomen [8]. Facial lesions similar to the one presented in this case have been reported previously [9,10]. Cutaneous lesions are frequently accompanied by metastatic disease at other sites, most commonly the lungs, bones, liver, and brain. Consequently, they are not candidates for curative therapy and have poor prognoses. The mean survival after diagnosis is around 11 months, and 96% of patients die within 36 months [7]. Therefore, treatment is usually supportive, with previous reports including palliative resection [8]. 

In this case, the patient reported a significant impact on his quality of life as the mass continued to bleed, leading to multiple hospital admissions. As a result, palliative surgical resection was ultimately offered. Interestingly, the patient reported two previous resections within a year of presentation with subsequent rapid regrowth. The data on recurrence of cutaneous metastases after surgical resection is limited, likely due to a lack of follow-up secondary to poor prognosis. One case reported successful treatment of a scalp nodule with Mohs surgery without recurrence in the following two years before the patient died of progressive disease at other sites [11]. In the case presented, the cause of recurrence is unclear but may have been due to incomplete resection in previous operations. More research into the recurrence of cutaneous metastases is needed to determine common causes and how to prevent them.

Additionally, the patient had known RCC metastasis to the bladder, a rare site for RCC. To the best of our knowledge, this is the first case of metastases to the bladder and cutaneous tissue reported together in a single RCC patient. These rare occurrences likely resulted from progressive disease in the setting of prolonged survival, as the patient presented more than five years after his initial RCC diagnosis. 

## Conclusions

Cutaneous and bladder metastases of RCC are uncommon, though several cases have been reported. Physicians should have a high suspicion of cutaneous metastases in RCC, as they may be the first sign of disease or systemic dissemination. An appropriate diagnostic workup should always be conducted. Unfortunately, the prognosis after diagnosis is generally poor. Palliative resection may improve the patient's quality of life, but ongoing and individualized surveillance is warranted, as regrowth can occur. Further research should focus on how multimodal approaches could be implemented in both the treatment of these metastases and the prevention of recurrence.
